# Environmental stewardship: A systematic scoping review

**DOI:** 10.1371/journal.pone.0284255

**Published:** 2024-05-07

**Authors:** Lynette J. McLeod, Jane C. Kitson, Zack Dorner, Natasha A. Tassell-Matamua, Philip Stahlmann-Brown, Taciano L. Milfont, Donald W. Hine

**Affiliations:** 1 School of Psychology, Speech and Hearing, University of Canterbury, Christchurch, New Zealand; 2 Kitson Consulting Ltd, Invercargill, New Zealand; 3 Waitaha, Kāti Māmoe, Ngāi Tahu; 4 Waikato Management School, University of Waikato, Hamilton, New Zealand; 5 School of Psychology, Massey University, Manawatū, New Zealand; 6 Te Ātiawa, Ngāti Makea kei Rarotonga; 7 Manaaki Whenua—Landcare Research, Wellington, New Zealand; 8 School of Psychology, University of Waikato, Hamilton, New Zealand; Swedish University of Agricultural Sciences and Swedish Institute for the Marine Environment, University of Gothenburg, SWEDEN

## Abstract

Environmental stewardship is a term describing both the philosophy and the actions required to protect, restore, and sustainably use natural resources for the future benefit of the environment and society. In this paper, we review the environmental science literature to map the types of practical actions that are identified as ‘environmental stewardship’ using the Preferred Reporting Items for Systematic Reviews and Meta-Analyses guidelines for scoping reviews. We specifically mapped: 1) the type of actions and outcomes targeting the natural environment that have been categorized as environmental stewardship, 2) the main actors, and the underlying factors influencing their environmental stewardship actions, and 3) the methods used to mobilize environmental stewardship actions once these factors are known. From the 77 selected articles, we found the term environmental stewardship encompassed a multitude of different actions, undertaken by a range of actors and addressing an array of issues that impact biodiversity on the land and in the water. These stewardship actions were conducted on both privately-owned and publicly managed lands and waterways, and across rural and urban landscapes. Despite many studies identifying characteristics and underlying behavioral factors that predicted actors’ participation in stewardship actions, there were few studies formally evaluating interventions to increase stewardship. Our review highlighted the term environmental stewardship is not embraced by all and is viewed by some as being inconsistent with aspects of indigenous worldviews. A better understanding of the concept of environmental stewardship and continued practical research into its practice is fundamental to empowering people to demand and enact environmental stewardship as well as for evaluating the success of their actions.

## Introduction

Growing pressures of climate change, land degradation and urbanization have heightened social and political attention to and investment in environmental management worldwide [1–4]. Underpinning the social and political responses to these problems is the concept of environmental stewardship, a term used for describing both a philosophy / ethic as well as the actions or behaviors required to achieve those aspirations [5–8]. Although there is no single authoritative definition of environmental stewardship in the literature, it is generally conceptualized as a broad, universal responsibility of humanity to care for the planet, to ensure that it can continue to provide the essential natural resources for life (refer to Table 1). It is considered a useful general term for describing actions that provide for natural environmental care and is entrenched within environmental policy and sustainable development in many industrialized nations [9, 10]. In this paper, we review the environmental science literature to map the types of practical actions that are identified as ‘environmental stewardship’, and how these are encouraged.

**Table 1:** Examples of the definitions for environmental stewardship from the literature.

Definition	Reference
“*Stewardship is a philosophy and approach to the care of land and ecosystems*, *a philosophy laden with the values of long-term health for ecosystems and the communities that depend on them*. *Stewardship may be practiced by individuals*, *communities*, *companies*, *organizations*, *and governments–singly or together*.”	Ack et al. 2001 [11, p. 119]
“*A popular term for the principles and actions aimed at improving sustainability and resilience of social-ecological systems at various scales and in different contexts*.”	Barendse et al. 2016 [12, p. 1]
“*The actions taken by individuals*, *groups or networks of actors*, *with various motivations and levels of capacity*, *to protect*, *care for or responsibly use the environment in pursuit of environmental and/or social outcomes in diverse social-ecological contexts*.”	Bennett et al. 2018 [5, p. 599]
“*Strategy to respond to and shape social–ecological systems under conditions of uncertainty and change to sustain the supply and opportunities for use of ecosystem services to support human well-being*.”	Chapin et al. 2010 [7, p. 241]
“*Responsible use and care of nature*, *and a ‘balancing act’ between stewards’ use of natural resources for agricultural production and their responsibility to protect and manage the wider ecosystem*.”	Cockburn et al. 2019 [13, p. 59]
“*The responsible provision of private good benefits to landholders for the delivery of long term public good ecological benefits to society that are sensitive to the landscape scale of ecosystem function*, *while encouraging collaborative conservation action across the private property boundaries of affected actors*.”	Cooke & Moon 2015 [14, p. 155]
“*The efforts to create*, *nurture and enable responsibility in landowners and resource users to manage and protect land and natural resources*.”	Mitchell & Brown 1998 [15, p. 8]
“*Responsible management of human activity affecting the natural environment to ensure the conservation and preservation of natural resources and values for the sake of future generations of human and other life on the planet*, *together with the acceptance of significant answerability for one’s conduct to society*.”	Welchman 2012 [16, p. 303]
“*A popular term for describing action in pursuit of sustainability*.”	West et al. 2018 [17, p. 30]
“*The responsible use (including conservation) of natural resources in a way that takes full and balanced account of the interests of society*, *future generations*, *and other species*, *as well as of private needs*, *and accepts significant answerability to society*.”	Worrell & Appleby 2000 [18, p. 263]

**Fig 1:**
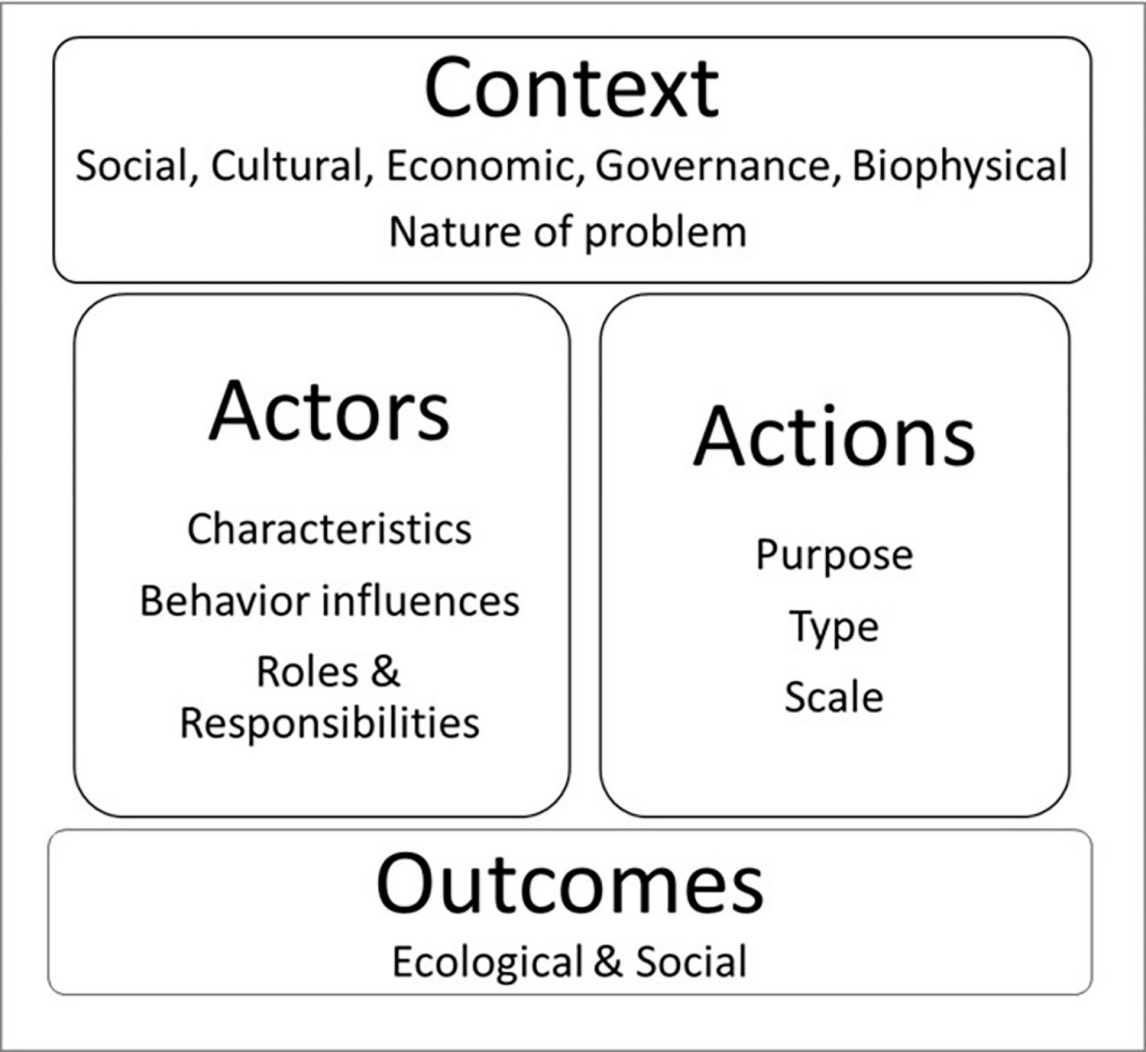
The four main dimensions of environmental stewardship as summarized from the literature.

There are four main dimensions to consider when operationalizing environmental stewardship: context, actors, actions, and outcomes (see Fig 1). Context sets the backdrop and boundaries for stewardship efforts [5, 19] and comprises the social, cultural, economic, biophysical or governance features operating in each situation. Collectively, these factors are key determinants of the normative dimension of environmental stewardship, conveying to actors what is appropriate and feasible [20]. The desired end-product of environmental stewardship efforts are the positive outcomes such as protection of wildlife and plant species, conservation of ecosystems, restoration of degraded habitats and sustainable use of natural resources and link to desirable social outcomes such as water and food security, health and well-being, employment and livelihoods [5, 8, 17, 21].

People performing stewardship actions can act either individually or collectively within a group. The identity of actors and their roles and responsibilities depends on the context, the nature and location of the problem being addressed and the purpose of the environmental stewardship actions. The ability and willingness of the actors to participate is influenced by their characteristics, along with their capability, opportunity and motivation (behavioral influences) to participate [5, 8, 19, 22–27].

Environmental stewardship actions aim to protect, conserve, restore or sustainably use the natural environment. The purpose of the action will dictate the type of action required, selected from a range of approaches, activities, behaviors and technologies. For example, restoration of habitats may involve removing man-made structures or planting native plants whereas protecting an endangered wildlife species may involve controlling competing species or developing an off-site breeding program. Stewardship actions can occur at different scales, from local to regional and national to address issues of varying complexity and can be driven by a range of decision-making processes including bottom-up community-driven actions and top-down mandatory government policies [5, 11, 13, 28].

Stewardship actions targeting the natural environment can potentially cover a huge and complex range of approaches, activities, behaviors and technologies by a diversity of actors. In many countries however, the term is commonly associated with actions that target sustainable provision of ecosystem services, that is, benefits provided to humans from the transformation of natural resources into the flow of essential goods and services e.g., food, water, and clean air [29], as well as to secure biodiversity priority areas through agreements with rural landowners [e.g. 13, 14, 25, 30, 31]. This is mainly owing to the fact that privately-owned rural lands are not only one of the main providers of ecosystem services, but also can serve as important reservoirs of biological diversity [29]. In addition, agricultural practices used to produce food and other resources are also considered a major contributor to biodiversity loss, with the conversion of natural habitats to intensely managed systems and the release of harmful chemicals and pollutants [32]. Participation in stewardship actions in these rural areas are generally mobilized using a range of market-based instruments, such as subsidized agri-environmental or payment-for-environmental services schemes and voluntary conservation programs [e.g. 30, 33, 34]. Less clear, however, is how the concept of environmental stewardship is applied in other contexts such as public lands and waterways, and in urban areas where the largest proportion of people reside, and how associated stewardship actions are mobilized.

A scoping review is a useful tool for outlining the key concepts underpinning a research area, mapping the main sources and types of evidence available and identifying research gaps in the existing literature [35–37]. The goal of this systematic scoping review is to establish how environmental stewardship has been operationalized to address natural environmental outcomes and examine its nature and associated characteristics. In conducting this review, we had three objectives:

1. What type of actions and outcomes targeting the natural environment have been categorized as environmental stewardship?
2. Who are the main actors, and what are the underlying factors that influence their environmental stewardship actions?
3. How are environmental stewardship actions mobilized once these factors are known?

## Methods

This systematic scoping review followed the Preferred Reporting Items for Systematic Reviews and Meta-Analyses guidelines for scoping reviews (PRISMA-ScR) [38].

### Data sources and search strategy

A search of the international literature using the keywords of ‘environmental stewardship’ was conducted to collect the broadest scope of information possible. This search was completed between April and May 2021. To gain a broad coverage of the peer-reviewed literature across multiple disciplines, we searched a range of information sources, including Web of Science, PsycINFO, CAB abstracts, SCOPUS, Science Direct and Google Scholar. Searches were undertaken in the English language only. After an initial scoping trial, the following combination of keywords was used: (“environmental steward*”) AND (conservation OR sustainability OR “resource management” OR motivation OR biodiversity OR indigenous OR “human dimension”) where *indicates a wildcard. In search engines that lacked the wild card ability, the terms “steward OR stewardship” were used. All retrieved records were saved into an EndNote database.

The initial search yielded 2482 records; however, 892 duplicate records were removed prior to assessment. All saved citations were then examined at the title and abstract level by a single reviewer. A second reviewer examined a random subset of 25% of the citations separately to provide indication of inter-rater reliability based on the eligibility criteria described below (Cohen’s Kappa test: K = 0.824). Articles were accepted as relevant to the next stage of the review process (full text assessment) if they appeared to contain information relevant as described in the eligibility criteria below. Acceptance into the review after the full text assessment stage was divided between the same two reviewers who worked independently.

### Screening and eligibility criteria

Explicit inclusion criteria were defined prior to screening of abstracts and full texts. To be eligible to be included in this review, an article needed to:

- Relate to the operationalization of ‘environmental stewardship’ targeting natural environment topics (i.e., use these terms in their title or as keywords to describe their content); and either,
- Identify any actions that are undertaken to achieve ‘environmental stewardship’ outcomes, and / or
- Identify any empirical outcomes of any actions undertaken including any identified
  ○ actors and their relationships
  ○ factors influencing these actions
  ○ description of how actions were mobilized

**Fig 2:**
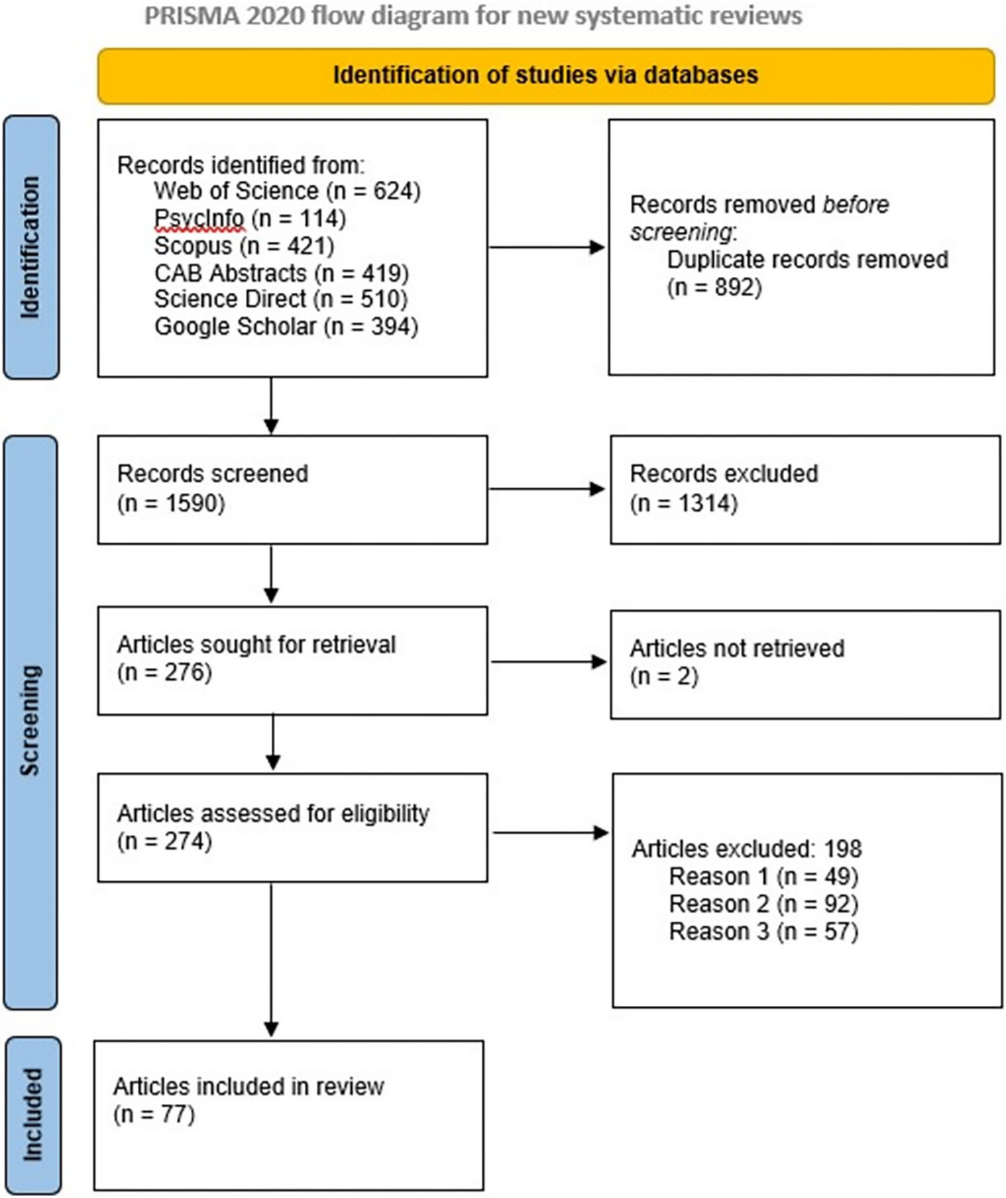
Adapted Preferred Reporting Items for Systematic Reviews (PRISMA) flow diagram indicating search and screening process for the present literature review [after 39].

Fig 2 summarizes the search and screening process according to the PRISMA guidelines. The reasons for exclusion were:

- Reason 1 –Studies outside the natural environment context (e.g., mining or health-related contexts)
- Reason 2 –Conceptual-type articles without evidence of identified actions or empirical outcomes
- Reason 3 –Policy-type articles without evidence of identified actions or empirical outcomes.

## Results

### General characteristics of literature included in the review

The systematic scoping search resulted in 77 peer-reviewed articles. These articles are summarized in Table 2 (articles that investigated the factors that influenced participation in environmental stewardship actions) and Table 3 (articles that evaluated interventions designed to mobilize action).

**Table 2:** Identification of factors influencing environmental stewardship actions from the selected articles.

Authors	Year	Actors	Actions	Influential factors
*Outcome*: *Protection*, *conservation and/or enhanced ecosystem services (natural resource management) on privately-owned lands*				
Addison & Pavey	2017	Rural landowners	Conduct actions to benefit small native mammals (e.g. predator control)	Environmental values and relations with implementers of control (trust, shared goals, collaboration, shared learning and acknowledgement of landowner’s knowledge
Ahnström et al.	2009	Farmers	Participation in conservation practices	Attitudes towards viability of actions, farming context, circumstances, location, interaction with agri-environmental scheme
Atari et al.	2009	Farmers	Participation in Environmental Farm Plan program	Farm income, years of farming experience, type of agribusiness, publicising of stewardship practices, use to help improve relationships with non-farming neighbours, compliance with regulations
Bond et al.	2018	Rural landholders	Participation in conservation incentive programs	Type of landholder (owner-occupier, absentee or group)
Burke & Running	2019	Farmers	Adoption of soil and water conservation practices	Role identity, environmental attitudes
Chapman et al.	2019	Farmers	Enrolment in riparian buffer conservation incentive programs	Value conflicts: implication of program rules, aesthetic, active land management, parcel-specific and community knowledge
Cooke & Lane	2015	Non-farming rural landholders	Engagement in environmental management practices	Awareness, knowledge, experience
Cross & Franks	2007	Farmers and advisors	Participation in Environment Stewardships Schemes	Attitudes towards schemes, ease of application
Darragh & Emery	2018	Farmers	Continued participation with environmental behaviours following end of agri-environmental scheme	Crowding-out theory, intrinsic and extrinsic motivations
DeAngelo & Nielsen-Pincus	2017	Rural landowners	Participation in Payment for Watershed Services programs	Attitudes, trust, ecological understanding and technical capacity more important than financial considerations
Farmer et al.	2017	Rural landowners	Participation in voluntary conservation programs (e.g. removal of invasive species, control of erosion)	Environmental motives, family life, previous positive experiences
Floress et al.	2017	Landowners / farmers	Adoption of conservation practices for water quality	Awareness of water quality problems, environmental, economic and social attitudes
Greiner	2015	Pastoralists and graziers	Participation in biodiversity conservation agri-environmental scheme	Personal attitudes and motivations, contract preferences
Greiner	2016	Farmers	Participation in biodiversity conservation agri-environmental scheme	Contract attributes, conservation requirement, payment, contract duration and flexibility, land productivity and attitudes
Lobley & Potter	1998	Landowners	Participation in agri-environmental schemes	Design of scheme can influence type of farmer, either motivated by financial gain or conservation motives
Lute et al.	2018	Landowners	Participation in native grassland conservation program	Value of ecosystem service, practitioner-landowner relationships, complexity of program
Mills et al.	2018	Farmers	Voluntary unsubsidised environmental practices	Agronomic and environmental motivations more than financial
Raymond et al.	2016	Farmers	Participation in landscape management actions	Understanding of landscape stewardship and values (environmental, production, holistic or instrumental)
Schaible et al.	2015	Farmers	Participation in working land conservation and pest management practices	Farm, field, economic and environmental characteristics, and operator motivations
Tong et al.	2017	Landholders (producer and absentee)	Adoption of soil and water conservation practices	Reasons for adoption, attribute values, land tenure
***Outcome*: *Protection*, *conservation*, *restoration and / or sustainability of natural environment and resources on public lands and waterways***				
Akankali & Chindah	2011	Artisanal fishers	Adoption of fishery conservation measures	Age, education level, values, fishing experience, public enlightenment, regulatory pressure, severity of pollutants, economic circumstances, institutional support, information access and location
Alender	2016	General public volunteers	Participation in citizen science water quality monitoring	Age, sense of helping the environment and contributing to scientific knowledge
Bleam	2018	General public volunteers	Participation in conservation activities	Place meaning
Bramston et al.	2011	Rural environmental group members, concerned residents	Volunteering with groups or voicing environmental concerns	Sense of belonging, caretaking of the environment, expanding personal learning
Darkson et al.	2020	General public	Participation in pro-environmental marine actions	Age cohort, marine environment values
Ding & Schuett	2020	General public volunteers	Participation in wildlife organization’s programs	Motivations (helping environment, values, learning, career, social, organisation of programs), satisfaction (organizational support, sense of empowerment, group integration), commitment (affective, normative) and generativity (focus on next generation)
Ganzevoort & van den Born	2020	Nature volunteers	Participation in conservation work	Four types found–recorders, restorers, educators and administrators. Nuanced differences in motivations between types but all types had personal connection to nature and wanted to contribute to its conservation
Gottwald & Stedman	2020	Citizens	Adoption of environmental actions	Place relations, values, attitudes, capacity
Jerome et al.	2017	General public volunteers	Engaging in voluntary environmental programs	Status, location, timeframe, membership, activity focus, governance, communications, resources and recognition
Johnson et al.	2021	White water rafters	Participation in public land management reflecting tenets of Leave No Trace program	Place-based motivations, normative beliefs, involvement in outdoor recreation activities, activity-based motivations (be around similar people, enjoy nature, escape personal and social pressures)
Kainamu-Murchie et al.	2018	Indigenous Estuarine shellfishers / harvesters	Adoption of estuarine shellfishing practices that meet Ngāi Tahu ethic (ki uta ki tai)	Indigenous and local socio-cultural values, knowledge and experience about estuarine stressors
Landon et al.	2018	Anglers	Adoption of voluntary stewardship behaviours	Identification as an angler. Moral norms and beliefs
Maund et al.	2020	General public volunteers	Contribution of data to citizen science projects	Strongest motivators are intrinsic value for environment and want to support research, learn and gain knowledge
Merenlender et al	2016	General public volunteers	Participation in Naturalist programs	Motivations–learning about local environment, plant and animals, connecting with nature, becoming certified, spending time with similar people. Lack of time was a barrier
Moller et al.	2009	Indigenous seabird harvesters: Rakiura Māori	Compliance with Rakiura Māori customary conservation ethic and sustainable harvest of seabirds	Indigenous traditional pathways of learning (observing, hands-on experience and storytelling), awareness of ancestors (tupuna) and taboo and connection to harvesting islands enhance compliance. Modern needs and pressures threaten knowledge transmission between generations. Other barriers–modern processing, transport and communication techniques
Newberry & Israel	2018	General public	Participation in Natural Program	To learn, help environment, act on altruistic values
Peck et al.	2021	Recreationists (skiers, kayakers, walkers using public paths)	Taking better care of public goods (e.g. picking up litter, donations for maintenance)	Feelings of ownership and perceived responsibility
Raynal et al.	2020	Recreational fishers	Engagement with outdoor recreation conservation organizations	Pro-environmental attitudes, enthusiasm, knowledge about coastal marine ecosystems
Reo et al.	2017	Indigenous partners	Continued engagement with multi-actor regional partnerships/collaborations	Respect for Indigenous knowledges, control of knowledge mobilization, intergenerational involvement, self-determination, continuous cross-cultural education and early involvement
Ryan et al.	2001	General public volunteers	Continued participation in environmental programs	Helping environment and learning, social factors, project organization, experienced change in values and attitudes
Schild	2018	Outdoor recreationists	Volunteering with projects preserving and restoring recreational resources	Civic engagement, environmental values, identity/enduring involvement, social/career networking, personal learning and obligation
Schuett et al.	2014	Recreational anglers	Volunteering with fishing or conservation organizations	Motivation–helping environment, learning, meeting people and influencing policy
Strzelecka et al.	2018	Volunteer travellers	Participation in ecological restoration projects	Motivations–worthwhile activity, age, hedonic experience
Takase et al.	2019	Volunteers	Continued participation in conservation projects	Frequency of participation influenced by improvement of personal physical and mental well-being, interaction with other people, enjoyment of cultural services from ecosystem
Tuntivivat et al.	2018	Indigenous youths	Engagement in environmental sustainability projects	Personal values, attitudes, learning and lifestyles, exposure to environment
Turnbull et al.	2020	Local coastal residents	Engagement with local coastal environmental stewardship projects	Attraction to marine wildlife, self-identity as local, worldview, size of local network and norms of informal enforcement
***Outcome*: *Protection*, *conservation*, *and / or restoration of natural environment and resources in urban areas***				
Asah & Blahna	2013	General public volunteers	Participation in conservation activities	Motivations: desire to help environment, defend and enhance ego, career and learning opportunities, escape and exercise, social interactions, community building. Continued volunteering: personal, social and community more than environmental motivation
Asah et al.	2014	General public volunteers	Participation in restoration and conservation activities	Demographics, social motivations more than environmental reasons
Bharati & Mohamed	2013	Urban residents	Participation in neighbourhood park management	Motivations: sense of attachment, attitude, community values
Coleman et al.	2018	Residential landowners	Adoption of Green Stormwater Infrastructure (raingarden, infiltration trenches, active diversion of rain run-off) to reduce stormwater pollution	Range of spatial, social and physical factors. Onsite and neighbour stormwater problems motivated use of trenches. Diversion used by residents with tendency for ‘green’ behaviours
Enqvist et al.	2017	Local residents	Participation in urban waterfront and waterbody environmental projects	Place attachment, place meaning, group type (community, environmental or recreational)
Johnson et al.	2019	Urban environmental groups	Beneficial local environmental group actions	Number of groups dependent on organization landscape, socio-economic factors and environmental aspects
***Outcome*: *Nurture connection to nature and / or improve environmental knowledge to build stewardship values and capacity***				
Chase & Levine	2018	General public volunteers	Participation in natural resource monitoring programs	Specific environmental attitudes towards the natural resource and more general attitudes
Eilam & Trop	2014	Adults	Development of environmental attitudes and behaviour	Influence of school programs on parents, formative experiences, personality
Garcia-Martin et al.	2018	Residents	Improve perceptions towards landscape stewardship	Perception of landscape values, place attachment, awareness of consequences, personal responsibility and capabilities, socio-cultural context
Hood et al.	2011	Rural youths	Engagement in stewardship activities	Attachment to place, concern for local natural resources
Sen & Nagendra	2020	Lake visitors and workers	Improve interest in stewardship actions	Environmental and social placemaking (emotional attachment)

**Table 3:** Selected articles that evaluated environmental stewardship interventions.

**Authors**	**Year**	**Actors**	**Intervention**	**Results**
***Outcome*: *Increased participation in protection*, *conservation and/or ecosystem services on privately-owned rural lands***				
Adams et al.	2012	Private land managers	Financial stewardship program	Compared potential costs of required actions to financial payment. Estimated $AU1million/year needed to cover 90% of catchment area
Ambrose et al.	2006	Landowners and managers	Awareness and management program for riparian land	19% of participants learnt new information, 21% likely to implement change, 100% with frequent interactions learnt new information compared to 70% with little interaction. Diverse interactions led to altered management.
Czap et al.	2019	Farmers	Recruitment letters for participation in Conservation Stewardship program	Personalised letter with hand-written phrase better than personalised all standard typed which was better than photocopied letter and no letter
***Outcome*: *Increased participation in protection*, *conservation*, *restoration and / or resource management of natural environment on public lands***				
Pillemer et al.	2017	Older adult volunteers	Retirees in Service to the Environment program	Successful in recruiting new individuals and providing substantial hours of volunteer time to communities. Program satisfaction high and positive outcomes from participation
Popp et al.	2020	Indigenous locals	Indigenous Guardianship monitoring and management programs	Enhances monitoring and management initiatives with inclusion of indigenous participation, knowledge and local information
***Outcome*: *Enhanced connection to nature and / or environmental stewardship values*, *attitudes and capacity***				
Aguilar et al.	2008	3rd-5th grade students	School gardening program	Both treatment and control developed positive attitudes and locus of control related to environment. Differences between previous garden experiences, gender and ethnicity.
Andrejewski	2012	5th grade students	Outdoor school program	Treatment showed increase nature connections, ecological knowledge, stewardship behaviour compared with control
Baird et al.	2020	General public	Outdoor Experiential NOLS course	Affirms NOLS environmental ethic: connection to nature leads to environmental concern and protection which engenders positive pro-environmental intentions
Ballard et al.	2017	Youth	Coastal program and water quality monitoring program	Each project developed different aspects of environmental science agency (understanding of environmental science, inquiry practices, identification with these practices and developing belief in importance of ecosystem)
Cudworth	2020	Children	Forest School programs	Involvement promoted reconnection to nature, benefited well-being and promoted pro-environmental attitudes and behaviour
Ernst	2017	Families	Nature play programs at zoos and aquariums	Involvement helped overcome barriers to spending time in nature
Gallay et al.	2016	Rural students	Place-based stewardship education	Significant increase in environmental awareness and behaviours, community attachment, confidence for action and increase in commitment
Griffin et al.	2016	High school students	Wildlife Conservation Camp	Moderate to large increase in knowledge and conservation interest, major influence on course of life career choice
Merenlender et al	2016	General public volunteers	Naturalist programs covering weed management and habitat restoration	Participants increased knowledge about ecosystems, greater confidence in conservation and continued engagement in citizen science
Pitt et al.	2019	Secondary school students	Forest Service citizen science projects	Programs incorporating both science and environmental education created ecological literacy among participants
Powell et al.	2018	Youth (8–13 years old)	Junior Ranger program	Immediate influences om awareness, interest and cognitive engagement which has influence on intentions and behaviour
Schwass et al.	2021	Participants	Outward Bound expeditions	Positive association between exposure to nature and increased sense of connection and stewardship towards nature, change in values, lifestyle choice and inspire action to improve state of environment
Stern et al.	2008	Participants	Residential Environmental Education program	Positive short-term effects on connection to nature, interest in learning and discovery, awareness of biodiversity and conservation lands, need for stewardship. Larger courses enhances outcomes
***Outcome*: *Direct benefit to environment***				
Ramirez-Reyes et al.	2018	Landholders	Reduction in deforestation and forest fragmentation offered by Payment for Ecosystems Services	Payment reduced total area of deforestation and fragmentation but may have increased incentive for landholders to hide deforestation in ways that increased fragmentation and habitat loss
Still & Byfield	2010	Landholders	Protection of endangered plant species offered by Environmental Stewardship programs	Entry level program only had potential to protect 15% of plant species while higher level program had potential for 89% of species. Complex requirements of many rare plants outside of both programs scope

**Fig 3:**
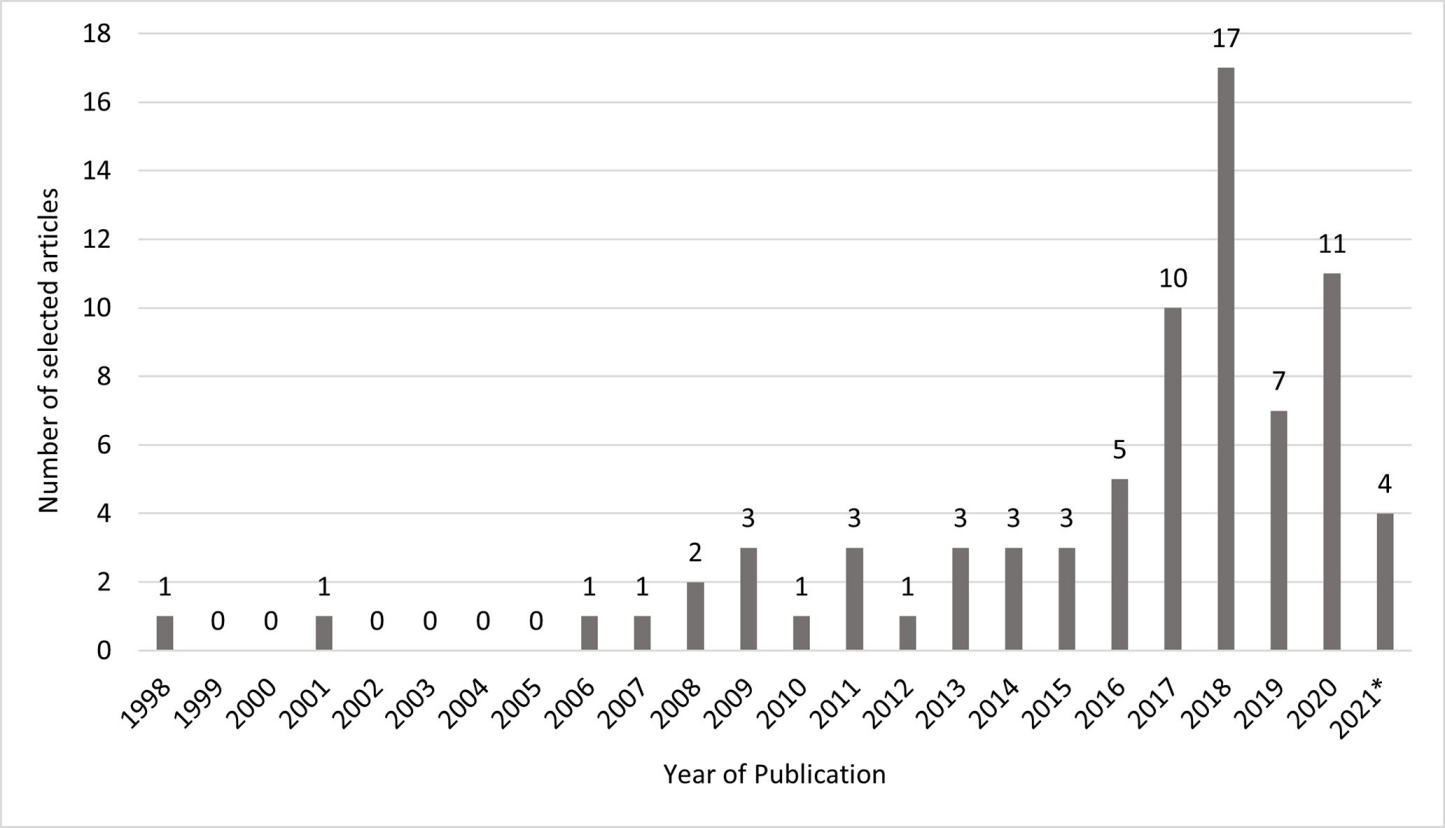
Number of selected articles per year identifying with ‘environmental stewardship’ (*only up to the May 2021 included).

The oldest selected article identifying as ‘environmental stewardship’ was published in 1998. However, it has only been in the last six years that this term has appeared more frequently in the literature (Fig 3). Studies originating from 13 countries were selected. The United States had the largest number of articles (*n* = 40), followed by Australia (*n* = 10), the United Kingdom (*n* = 9) and Canada (*n* = 6). India, New Zealand and Germany each had two articles and the remaining countries, Japan, Mexico, Netherlands, Nigeria, Philippines and Sweden each had one article.

### *Types of outcomes and actions categorized as* environmental stewardship

Multiple problems that were directly impacting the natural environment and stemming from or relating to resource mismanagement, environment degradation, urbanization and climate change were addressed in the selected articles. These included environmental problems from across both rural and urban landscapes, on the land (e.g. soil erosion, deforestation, wildlife survival) and in waterways and marine environments (e.g. water quality, sedimentation, marine life). Another problem addressed in the selected articles was related to an aspect indirectly impacting the environment–people’s lack of connectivity to nature. It is argued that developing both physical and emotional connections to nature helps shape environmental attitudes and foster future stewardship behaviors [e.g. 40–43]. Our articles are summarized under four main outcome categories (Tables 2 & 3):

- Protection, conservation, restoration and/or enhanced ecosystem services (natural resource management) of privately-owned rural lands,
- Protection, conservation, restoration and / or sustainability of natural resources on publicly-managed lands and waterways,
- Protection, conservation, and / or restoration of natural resources in urban areas,
- Nurture connection to nature and / or improve environmental knowledge to build stewardship values and capacity (stewardship capital).

Outcomes of environmental stewardship efforts across privately-owned rural landscape were to sustainably use, protect, conserve and restore a range of environmental resources found there, including water, soil, wildlife, forests and other vegetation [e.g. 44–47]. The outcomes for environmental stewardship efforts towards public natural resources, such as waterways, coastal and marine environments and public managed lands were to protect, conserve, restore or sustainably manage the resources [e.g. 48–51]. The outcomes for environmental stewardship efforts in urban areas were to protect, conserve or restore natural resources found in these areas [e.g. 52–54]. Outcomes for efforts to build stewardship capital were to nurture connection to nature, improve environmental knowledge and build stewardship values and capacity in both children and adults.

The selected articles described a range of actions to achieve their stewardship outcomes. For example, actions on productive rural lands to protect, conserve or sustainably use natural resources included control practices for weeds, predators and soil erosion, as well as modified practices for water use and forest clearing. Actions to protect, conserve, restore or sustainably use natural resources on public lands included adoption of sustainable fishing / harvesting practices, water quality monitoring, removing litter and planting native trees [e.g. 49, 51, 55, 56]. Actions to protect conserve or restore natural resources in urban areas included building a raingarden to reduce stormwater run-off, participating in neighborhood park management or local environmental group activities [e.g. 53, 54, 57]. Actions to build stewardship capital included participation in learning and experiential activities such as gardening, nature play, citizen science projects and environmental-focused travel [e.g. 43, 58–61].

The use of alternate keywords aside from environmental stewardship were evident, depending on the context of the article and the type of outcomes and actions described. In Indigenous contexts the terms guardianship or custodianship were used [62]. The Māori term Kaitiakitanga was used in specifically in articles referring to stewardship actions within Aotearoa New Zealand [51, 63]. In conservation contexts stewardship-type actions were also more specifically referred to as pro-environmental behaviors, conservation management, environmental preservation or ecosystem management [e.g. 41, 44, 49, 52, 60, 64–67]. In resource management contexts terms such as environmental or ecosystem services or natural resource management were also used [68, 69], and in urban contexts the term nature-based solution was applied. Some authors described specific types of stewardship which related to explicit contexts and actions, for example, landscape stewardship [70, 71], conservation stewardship [52], place-based stewardship [66], civic environment stewardship [72] and virtual stewardship [73]. Peck and co-authors [74] differentiated between effortful stewardship (which result from direct actions of participants such as picking up litter) and financial stewardship (where people make financial donations towards stewardship actions).

### Main actors and underlying factors influencing their actions

Environmental stewardship actions are conducted by people (or actors), either individually or in a group, and their he identity depends on the location of the problem being addressed and purpose of the environmental stewardship action. In urban areas and public natural spaces, actors included local community members, user groups and public volunteers, as well as the organizations who engage with them [e.g. 72, 74–77].

In rural areas, the main actors include private land managers, agricultural advisors, and government institutions [71, 78]. Private land managers can be categorized in a variety of ways, such as farmers / producers (on-farm or absentee), and non-producers (amenity migrants or life-stylers) [e.g. 41, 79, 80]. Environmental stewardship capital efforts involved actors ranging from pre-school, school-aged students and young adults through to senior adults [e.g. 58, 81–83].

Three quarters of the selected articles (74%, refer to Table 2) investigated actors’ traits and behavioral factors that influenced the adoption and participation in stewardship actions. A number of behavioral theories and concepts were used to inform research in this area, including the Theory of Planned Behavior [68], Place Attachment [77], Value-Action Gap [44], Motivation Crowding Theory and Crowding Out Theory [84] and the ‘good farmer’ concept [65]. A range of important factors were identified and are listed in Table 2 including actors’ characteristics, capacity, opportunities, values, and other motivations as well as social dimensions such as social-relational dynamics between actors, social equality and institutional pressures.

In rural areas, land managers’ perceptions of environmental stewardship influenced their adoption of appropriate stewardship behaviors [71]. Factors that were identified included perceived environmental benefits and viability of actions, improved knowledge and capacity, anticipated financial outcomes, flexible contract conditions and social benefits [69, 85–89]. Barriers to participation included awareness of problems or schemes, attitudes, lack of trust, value conflicts, ease of gaining entry into a particular scheme, cost, uncertainty of outcomes, types of farming enterprises, previous negative experiences and socioeconomic factors [45, 78, 90–94].

In marine and coastal (public natural resources) contexts, values, social norms, attraction to marine wildlife and self-identity as a local were related to participation in stewardship actions [50, 95]. Identity, values, norm beliefs, knowledge, age, education levels, economic circumstances, fishing experience and local environmental conditions were some of the factors that influenced the willingness of anglers to participate in fishery conservation actions [48, 64, 96, 97]. Emotional attachment to place, values and past experiences are important drivers for waterways stewardship actions [98, 99]. Those actors that rely on healthy environments for recreational activities were found to be more motivated to be better stewards due to their perceived ownership of a particular natural resource [74].

The main factors influencing participation in urban landscapes include emotional attachment to place [53, 77], social interactions, community building and desire to help the environment [52, 57] as well as group dynamics, composition and objectives [100]. The main motivations for volunteer participation included helping the environment or community, contributing to science knowledge, career and learning opportunities, social interaction, developing a sense of belonging, personal satisfaction and personal well-being [55, 56, 101–106]. These last four factors were found to be important motivators for repeated participation [107]. Younger adults tended to be motivated by hedonic experiences, gaining a sense of enjoyment, pleasure and excitement through consuming and exploring unique ecosystems [108].

To effectively engage the Indigenous community in external agencies natural resource management and stewardship strategies, two of the articles highlighted the importance promoting collaboration and incorporating the cultural and social mechanisms of local Indigenous communities while taking a more holistic view of the environment (as opposed to the current compartmentalized processes of most government policy) [51, 63]. Reo and co-authors [76] identified six factors important to keeping Indigenous partners engaged: 1) early involvement, 2) respect for Indigenous knowledges, 3) control of knowledge use, 4) intergenerational involvement, 5) self-determination and 6) continuous cross-culture education.

### Mobilization of environmental stewardship actions

The remaining quarter of the selected articles (26%, refer to Table 3) evaluated the effectiveness of interventions designed to either increase participation in environmental stewardship behaviors (5 articles), enhance environmental stewardship capital (13 articles) or improve the targeted environmental resources (2 articles). Early education, outdoor-school, naturalist programs and technology were considered important tools to enhance emotional connections to nature, shape environmental attitudes, and foster future stewardship behaviors. Increasing opportunities for young people to interact with nature was found to be important and influential, through the design of their learning and play spaces [40, 60], curriculum content [66] and offering a variety of out-of-classroom programs, such as school gardening [58] and outdoor schools, camps and programs [67, 82]. As children matured, place-based education [109] and real-world projects [110] as well as identifying future roles [73] were found to foster future environmental stewardship attitudes and behaviors. A wide range of programs have been explored for adults as well, including tertiary curriculum content [111], adult naturalist programs [42, 112, 113], zoo and aquarium education programs [81], outdoor experiential activities [41, 43] and volunteer / citizen science programs [105].

Market-based instruments, such as subsidized agri-environmental or payment-for-environmental services schemes and voluntary conservation programs to secure these stewardship actions [e.g. 46, 78, 114]. Methods to improve rural adoption and enhance these programs’ effectiveness include increasing subsidy amounts [64], using personalized recruitment letters [115] and improving interaction opportunities with awareness and management programs [92].

Two articles were selected that investigated the participation on stewardship actions on public natural resources. Popp and coauthors [62] highlighted the importance of including indigenous participation, knowledge and local information to enhance wildlife monitoring and management initiatives. Pillemer and coauthors [116] investigated the success of a conservation volunteer recruiting program. There were no articles selected that investigated interventions aimed at increasing participation in stewardship actions in urban areas.

## Discussion

This scoping review explored how environmental stewardship is operationalized in the environmental science literature, with a focus on its key characteristics–the type of outcomes and actions categorized as environmental stewardship, the main actors and the underlying factors that influence their actions, along with how this information has been used to mobilize actions. The findings are discussed below, along with the limitations of the current review, identified gaps in the literature and recommendations for future research.

### Outcomes and actions identifying as environmental stewardship and the main actors

The 77 articles selected in this review covered a range of stewardship actions and outcomes identifying as environmental stewardship. We found articles describing actions and outcomes that addressed the multitude of problems impacting the rural and urban landscapes, on land and in water, and on privately-owned and publicly managed lands. The term environmental stewardship was found to be used in the literature to describe these types of outcomes and actions for at least 25 years, becoming more prominent in the past six years.

A common focus of the environmental stewardship actions found in our selected articles was on managing, conserving and/or restoring natural resources and biodiversity in rural areas, targeting private landowners, producers and advisors. As the main providers of ecosystem services, as well as an important reservoir of environmental diversity [29], private rural landowners are considered key environmental stewards [32]. To secure environmental stewardship actions in these rural areas, many countries have introduced a range of market-based instruments such as subsidized agri-environmental or payment-for-environmental services schemes and voluntary conservation programs [31, 33, 117]. The large proportion of articles focusing on these schemes and identifying the factors that influenced land managers’ participation reflects the importance placed on this dimension of environmental stewardship in the academic literature [13, 18].

Another important focus of the selected articles was on stewardship actions to protect, conserve, restore and manage natural resources on publicly managed lands and in public waterways. Identified actions included monitoring water quality or wildlife populations, removing rubbish, participating in conservation or restoration activities with environmental groups and adopting sustainable fishing practices. The main actors were community members and organizations, users of recreational resources and harvesters of public resources (e.g. fish, shellfish). Community residents, visitors and environmental groups were the main actors targeted in environmental stewardship actions to protect, conserve and restore natural resources in urban areas. Identified actions included participation in conservation projects, neighborhood park management and adoption of stormwater pollution mitigation measures. These articles highlight the growing importance of the environmental stewardship concept and practice in the environmental management and conservation science literatures and policy [19, 118].

### Mobilization of environmental stewardship actions

In the past few decades, researchers in fields such as psychology, public health and environmental management have increasingly focused on examining the relationship between people and nature [119, 120]. Results have highlighted that people’s loss of connectivity to the natural environment not only has harmful effects on their health and well-being but also negatively influences their environmental concern and behavior [121, 122]. This is seen as an important behavioral influencer of stewardship and actors, and hence many of our selected articles [e.g. 40–43] focused on the development of both physical and emotional connections to nature, with the aim of shaping environmental values and attitudes, building stewardship capital. This is seen as a first step to fostering future intentions for stewardship behaviors and mobilizing stewardship actions.

The implementation of effective interventions to encourage and enable stewardship behaviors is critical for stewardship actions to be successful. An important component to developing interventions is to gain an understanding of the targeted audience’s characteristics and behavioral factors that influence participation [e.g. 123–125]. Three quarters of our selected articles focused on identifying the behavioral factors and actor characteristics that influenced their adoption and participation in particular stewardship actions. Different characteristics and behavioral factors were identified for specific actors and actions; for example, identified factors influencing participation in urban environmental stewardship actions included a person’s emotional attachment to place [53, 77], their social connections and their desire to help the environment [52, 57]. In contrast, factors influencing participation in rural stewardship actions included types of farming enterprises, farmers attitudes to the environment, awareness of problems, ease of implementing an action, perceived cost and related environmental benefits and trust in the organization implementing the actions [e.g. 45, 69, 78, 93].

Although many of the reviewed papers identified characteristics and behavioral factors that influenced participation in stewardship actions, very few described the actual behavior change tools used to mobilize these actions (aside from the agri-environmental incentive schemes) as well as evaluating the design, applicability and effectiveness of interventions. It is often expensive, both in terms of time and resources, to develop and implement behaviour change intervention to increase stewardship actions. Thus, it is important to rigorously assess the extent to which interventions positively impact stewardship actions other valued ecological or social outcomes. Without well-designed and competently implemented evaluations, it is difficult to assess the effectiveness of interventions and how future efforts might be improved [124]. This is unsatisfactory but presents an opportunity for future research to advance the knowledge on human behavior change and provide practical feedback to practitioners and policymakers.

### Indigenous perspectives

The meaning of environmental stewardship has evolved over time. Some have questioned the usefulness of the term and criticized it for being too rooted in religious thought [9, 18], inherently sexist, speciesist and centered around western worldviews that the natural environment is a resource that can be owned and exploited [16, 126, 127]. These stewardship relationships that have a notion of ownership of resources are inconsistent with many Indigenous worldviews, which have been developed and maintained collectively for centuries, and not only describe a duty of care for ecosystem management but also encompass the interconnectivity, reciprocity and relations of balance between all natural beings [128–133]. Over the past thirty years, there has been an increased inclusion of Indigenous worldviews and knowledge-holders when developing and planning local and national conservation / environmental actions [134–136]. Some communities are turning to Indigenous knowledge and experiences to provide insights, for example landscape management in Australia [137, 138], fisheries and waterway management in Canada [136, 139] and Aotearoa New Zealand [140–145]. However, there is still a gap in the recognition and support given to Indigenous stewardship and Indigenous knowledge in the scientific community and government policy [137, 146–152].

In Aotearoa New Zealand, statutes stipulate that the Indigenous Māori are included in environmental and conservation management, and there is an imperative to ensure the recognized *tāngata whenua* (Indigenous people of the country) are involved in decisions related to the environment. Māori are strongly connected with te taiao (the natural environment), and their cultural identity is rooted in their relationship with their landscape. This affiliation comes with inherited responsibilities from their ancestors and obligations to future generations [152–154]. The term ‘kaitiakitanga’ was first defined in the Resource Management Act 1991 as meaning “*the exercise of guardianship by the tāngata whenua of an area in accordance with tikanga Māori in relation to natural and physical resources; and includes the ethic of stewardship*” (Note: *tikanga Māori* refers to Māori customs and traditional values). This term has been used as a vehicle for applying ideas about guardianship, preservation, conservation, repair and use of the environments based on traditional Māori worldviews in Aotearoa New Zealand. However, traditional concepts akin to the contemporary use of the term kaitiakitanga encompass more than the notion of ‘guardianship’ and include a nuanced understanding and expression of the deep relationship between the spiritual realm, humans and the natural world [130, 153–155]. There is no single Māori perspective of this concept, and with the increasing use of this term in relation to conservation and environmental management in Aotearoa New Zealand [156], there is a strong need to understand what kaitiakitanga means to Māori, informed by the different iwi (Māori community), hapū (tribal) and whānau (family community) backgrounds of those involved.

### Limitations

This review has several limitations. Only existing peer-reviewed research that was published in English and available online was explored. This narrow search field may have led to the exclusion of relevant articles published in other languages or in government and organizational reports and non-peer-reviewed conference papers − all of which could result in susceptibility to publication bias [157, 158]. As is consistent with a scoping review methodology, we did not appraise the quality of selected articles as for a full systematic review [35–37], which should also be kept in mind when interpretating the findings. Finally, the results obtained by this review may have been limited by the search terms that were used. Although we did consider the convergence between environmental stewardship and related constructs such as conservation, sustainability, and resource management, other related terms used in specific contexts such as ecosystem management or nature-based solutions, were not included. This could have resulted in the exclusion of articles examining the constructs related to environmental stewardship, but not necessarily categorized as such.

## Conclusions

This scoping review revealed that there are multitude of different actions, undertaken by a range of actors identifying as ‘environmental stewardship’ in the literature. These stewardship actions aimed to achieve a variety of ecological and social outcomes on both privately-owned and publicly managed lands and waterways, across rural and urban landscapes. Their objectives were to protect, conserve or restore wildlife populations, habitats and ecosystems or sustainable use nature resources. Most of the studies selected in this review focused on identifying characteristics and underlying behavior factors that influenced actors’ participation in stewardship actions and many described how this information could be used to mobilize actions. However, our review also highlighted the paucity of literature in the subsequent description, design and evaluation of interventions in successfully mobilizing actions, as well the resulting benefits, or not, of these actions on the environment. Our review also highlighted, that even though the environmental stewardship plays in central role in guiding environmental policy in many developed nations, the concept is not embraced by all and is viewed by some as being inconsistent with aspects of indigenous worldviews. A better understanding of the concept of environmental stewardship and continued practical research into its practice is fundamental to empowering people to demand and enact environmental stewardship as well as for evaluating the success of their actions.

## Supporting information

S1 Checklist PRISMA 2020 for abstracts checklist. (DOCX)

S2 Checklist PRISMA 2020 checklist. (DOCX)
